# Long-term personal air pollution exposure and risk for acute exacerbation of idiopathic pulmonary fibrosis

**DOI:** 10.1186/s12940-021-00786-z

**Published:** 2021-08-30

**Authors:** Ioannis Tomos, Konstantina Dimakopoulou, Effrosyni D. Manali, Spyros A. Papiris, Anna Karakatsani

**Affiliations:** 1 2nd Pulmonary Medicine Department, National and Kapodistrian University of Athens, Medical School, “ATTIKON” University Hospital, 1, Rimini street, 12462 Haidari, Greece; 2 Department of Hygiene, Epidemiology and Medical Statistics, National and Kapodistrian University of Athens, Medical School, Athens, Greece

**Keywords:** Idiopathic pulmonary fibrosis, Air pollution, Acute exacerbation of idiopathic pulmonary fibrosis, Ozone, PM, Personal exposure

## Abstract

**Background:**

Urban air pollution is involved in the progress of idiopathic pulmonary fibrosis (IPF). Its potential role on the devastating event of Acute Exacerbation of IPF (AE-IPF) needs to be clarified. This study examined the association between long-term personal air pollution exposure and AE- IPF risk taking into consideration inflammatory mediators and telomere length (TL).

**Methods:**

All consecutive IPF-patients referred to our Hospital from October 2013-June 2019 were included. AE-IPF events were recorded and inflammatory mediators and TL measured. Long-term personal air pollution exposures were assigned to each patient retrospectively, for O_3_, NO_2_, PM_2.5_ [and PM_10_, based on geo-coded residential addresses. Logistic regression models assessed the association of air pollutants’ levels with AE-IPF and inflammatory mediators adjusting for potential confounders.

**Results:**

118 IPF patients (mean age 72 ± 8.3 years) were analyzed. We detected positive significant associations between AE-IPF and a 10 μg/m^3^ increase in previous-year mean level of NO_2_ (OR = 1.52, 95%CI:1.15–2.0, *p* = 0.003), PM_2.5_ (OR = 2.21, 95%CI:1.16–4.20, *p* = 0.016) and PM_10_ (OR = 2.18, 95%CI:1.15–4.15, *p* = 0.017) independent of age, gender, smoking, lung function and antifibrotic treatment. Introduction of TL in all models of a subgroup of 36 patients did not change the direction of the observed associations. Finally, O_3_ was positively associated with %change of IL-4 (*p* = 0.014) whilst PM_2.5_, PM_10_ and NO_2_ were inversely associated with %changes of IL-4 (*p* = 0.003, *p* = 0.003, *p* = 0.032) and osteopontin (*p* = 0.013, *p* = 0.013, *p* = 0.085) respectively.

**Conclusions:**

Long-term personal exposure to increased concentrations of air pollutants is an independent risk factor of AE-IPF. Inflammatory mediators implicated in lung repair mechanisms are involved.

**Supplementary Information:**

The online version contains supplementary material available at 10.1186/s12940-021-00786-z.

## Background

Idiopathic pulmonary fibrosis (IPF) represents a chronic and irreversibly progressive interstitial lung disease with poor prognosis [1]. During its course, many patients develop a sudden acute respiratory deterioration, of unknown cause, associated with high mortality. This event is referred as acute exacerbation of IPF (AE-IPF) and is characterized by the development of diffuse alveolar damage (DAD) upon usual interstitial pneumonia (UIP) [2, 3]. Even though diverse trigger factors have been proposed to contribute to the appearance of these catastrophic events, the exact etiology of the majority of the cases remains unknown [2, 3].

Recently, the contribution of air pollution exposure in IPF progress has emerged [4–13]. Although it is widely accepted that air pollution deteriorates diverse chronic respiratory diseases such as asthma and chronic obstructive pulmonary disease [14–16], there is lack of evidence on its potential role on the course of IPF and particularly on the devastating event of AE-IPF. Air pollutants could act as an environmental precipitant factor contributing to repetitive epithelial injury and leading thus, to aberrant wound healing [5]. Cumulative exposure through inflammatory pathways and oxidative stress could further contribute to the appearance of AE-IPF [5, 17, 18]. Studies have shown that early on upon the development of AE-IPF, triggered by other etiologies such as infection, aspiration and gastro-esophageal reflux, the inflammatory process seems to be initiated by a complex network of cytokines, among which IL-6 and the neutrophil chemotactic factor IL-8 play a major role both in progression and outcome [19, 20].

Based on the above, we hypothesized that air pollution could represent an additional environmental harmful stimulus capable to amplify lung injury and fibrotic processes, aggravating thus the outcome of this disease and leading to AE-IPF.

From the recent literature there is evidence that air pollution is associated with TL shortening, a factor well-known to be related with acceleration of biological aging and susceptibility to IPF [21–24]. Telomeres are DNA–protein structures that protect chromosome ends and preserve genetic information. As they shorten in each cell division leading finally to apoptosis and cell cycle arrest after reaching a critical point, TL could affect the association of air pollution and AE-IPF representing a modifying factor important in the pathogenesis and progress of IPF [25, 26].

The aim of the present study is to investigate the association between long-term personal urban air pollution exposure [ozone (O_3_), nitrogen dioxide (NO_2_), particles with a 50% cut-off aerodynamic diameter of 10 mm (PM_10_), particles with a 50% cut-off aerodynamic diameter of 2.5 mm (PM_2.5_)] and AE-IPF. Moreover, to delineate the underlying mechanisms, we examined the potential association of air pollution exposure with inflammatory mediators in the peripheral blood of IPF patients. Finally, to minimize potential confounding from TL we performed separate analyses in a subgroup of patients with measured TL ratio.

## Methods

### Study population

All consecutive IPF patients referred to our Pulmonary Medicine Department from October 2013 to June 2019 were included. Disease diagnosis was made according to the American Thoracic Society/European Respiratory Society/Japanese Respiratory Society/Latin American Thoracic Association consensus criteria after applying a multidisciplinary approach for each case [1]. Patients presenting AE-IPF during the study period were considered cases. They fulfilled all the proposed criteria of the international guidelines including worsening of dyspnea within the last 30 days, exclusion of identifiable causes, while any new ground glass opacities or consolidations were confirmed by new chest computed tomography (CT) at the time of admission [1, 3]. We also excluded deteriorating IPF patients secondary to cardiac insufficiency after performing echocardiogram and measuring N-terminal-pro brain natriuretic peptide levels in all exacerbated patients. The study protocol was approved by the local Ethics Committee (ΕΒΔ201/23–4-14, ΕΒΔ2720/04–10-2017) and participants provided written informed consent.

### Clinical data

Information on demographics, medical history and smoking status were collected for all participants. Clinical and laboratory findings including pulmonary function tests, time since first diagnosis, comorbidities as well as data on any immunosuppressive and/or antifibrotic treatment were recorded. In all patients hospitalized for acute exacerbation, computed tomography pulmonary angiogram (CTPA) was performed to exclude pulmonary embolism. Blood, sputum and bronchoalveolar lavage (BAL) cultures, when feasible, were performed to exclude any infection.

### Air pollution exposure assessment

We estimated personal long-term exposure to air pollutant concentrations for each patient, based on geo-coded residential addresses. For this purpose, we used maps with predicted annual average of O_3_, NO_2_, PM_10_ and PM_2.5_ in Greece for 2012, provided by the Ministry of Environment and Energy. The available air pollution maps are based on models applied to estimate air pollution concentrations nation-wide. Details of the procedure can be found in Progiou and Ziomas [27]. Briefly, the prediction method was based on the source apportionment estimation from the application of 3D meteorological and dispersion modeling. The air pollution measurements obtained from the fixed air pollution stations run by the Ministry of Environment provide data for all pollutants from 2002 and onwards. The models were validated by selecting sites with both predicted and measured air pollutant levels and evaluated by assessing their level of agreement and correlation. The validation procedure resulted in a correlation of 95.5%. The difference between predicted and measured concentrations for the whole validation period (2002 – 2012), ranged from − 7 to 7%.

### Inflammatory mediators’ measurements

Inflammatory mediators (IL-1a, IL-1b, IL-4, IL-5, IL-6, IL-8, IL-10, IL-13, active TGF-β, TNFa, CCL2, CCL3, CCL-18, MMP-1, MMP-7, MMP-9 and osteopontin) were measured by an enzyme-linked immunoabsorbent assay (ELISA) using Milliplex kits according to the manufacturer's instructions (Millipore, Billerica, MA, USA). The above mentionned proteins were analyzed on a Luminex 200 platform (Luminex Corp, Austin, TX, USA).

### Blood collection-DNA extraction-Telomere length measurement

In a subgroup of 36 IPF patients, a blood sample of 5 mL was collected from patients using the standardized phlebotomy. Directly after the collection, specimens were used for DNA extraction, using the QIAamp DNA Blood Mini Kit (Qiagen, Heidelberg Germany). DNA concentration was measured by QIAexpert (Qiagen, Heidelberg Germany) and only good-quality DNA with an A260/A280 ratio of 1.7–2.0 stored long-term in TE at -20 °C for further experimental procedures. Telomere length of genomic DNA from circulating leukocytes was determined using a multiplex quantitative polymerase chain reaction (qPCR) method as described by Cawthon [28]. Relative quantification is calculated by dividing the telomeric DNA product (T) by the reference gene (S) that is present as a single copy in the genome, to generate a (T/S ratio). All the experiments were carried out at the Rotor-Gene Real Time PCR cycler (Qiagen, Heidelberg Germany).

### Statistical analysis

We applied chi-square test and independent samples t-test for assessing differences in personal, health characteristics and exposure between patients with and without telomere length ratio measurements. The latter was also done to assess differences between patients residing within or out of the Greater Athens Area (urban vs suburban areas). We applied multiple logistic regression models to determine the relationship of O_3_, NO_2_, PM_10_ and PM_2.5_ and risk of AE-IPF. The outcome AE-IPF (indicator variable: no vs yes) was used to express whether a participant had at least one AE-IPF event during the study period. In the models, we adjusted for sex (female vs male), age (years), smoking status (3 dummy variables: non-smokers, ex-smokers, smokers), recent FVC (%) and DLCO (%) while in stable condition as well as antifibrotic therapy (yes vs no). Moreover, we applied 2-pollutant models when investigating the effect of O_3_, by also adjusting for PM_2.5_ personal long-term exposure. We conducted the analysis separately by residence (patients residing in urban or suburban area). We applied sensitivity analysis by: 1) including only patients with telomere length ratio measurements; 2) by including alternatively as an additional independent variable in the models i. annual mean temperature (assigned from nearest monitoring station to participants’ residence) and ii. job (blue vs white collar) as an indicator for socio-ecomonic status. To account for acute fluctuations of air pollution in risk for AE-IPF we also adjusted for distance to major road (m).

We applied multiple linear regression models to investigate the association of long-term personal exposure to predicted air pollutant concentrations and inflammatory mediators. Since the distribution of the inflammatory mediators was skewed (dependent variables) we log-transformed the variables. The analysis was done separately: stable and AE-IPF patients. In all models we adjusted for age, sex, smoking status and antifibrotic therapy. We addressed multiple testing bias by conducting Bonferroni *p*-value correction, thus by dividing the level of significance (α = 5%) by the number of inflammatory mediator outcomes (*n* = 17). Therefore, a result was statistically significant if *p*-value < 0.003.

Results from multiple logistic models are reported as Odds Ratios (OR) and corresponding 95% Confidence Interval (C.I.) per 10 μg/m^3^ increase in exposure. Results from multiple regression models are reported as % change (due to log-transformed dependent variable) and corresponding C.I. per 10 μg/m^3^ increase in exposure.

All epidemiological analyses were performed using the Stata/SE 10.0 for Windows statistical package (Stata CorpLP Lakeway Drive College Station, Texas, USA.). Geo-coding of patients’ residence and GIS analysis for prediction of personal exposure to air pollutant concentrations was done via ArcGIS Desktop, Release 10 (ESRI, 2011).

## Results

Demographic and clinical characteristics of the study subjects are presented in Table 1. In total, 118 IPF patients were included in the study [mean age ± SD (72 ± 8.3) years] with a male preponderance (74.6%). Fifty-five (47.4%) presented at least one acute exacerbation. None of the patients received immunosuppressive therapy during AE-IPF whilst all received broad-spectrum antimicrobial coverage (data not shown). Telomere length ratio was measured in a subgroup of 36 IPF patients [mean ± SD 0.79 (0.35)]. The coefficient of variation (CV) for telomere length ratio measurement is 44.2%. Patients with measured TL had higher most recent mean FVC value (79.5 ± 18.8 vs 67.3 ± 19.8, *p* = 0.004) than those without measured TL. However, they did not differ regarding the GAP stage, denoting that there was no significant difference in mortality risk (*p* = 0.425). Moreover, TL was not associated with age (*r* = -12, *p*-value = 0.499) and gender (males: mean, SD: 0.74 (0.29) vs females: mean, SD: 0.95 (0.48); *p*-value = 0.119).

**Table 1:** Demographic, functional, clinical and laboratory characteristics for all patients and for those with telomere length ratio measurement

**Personal & health characteristics**	**All patients** **(*n* = 118)**	**Patients with telomere length ratio (*n* = 36)**	**Patients without telomere length ratio (*n* = 82)**	***p*-value**^**a**^
Gender (male; n, %)	88 (74.6)	27 (75.0)	61 (74.4)	0.944^1^
Age (years; mean, SD)	72 (8.3)	72 (8.0)	72 (8.4)	0.886^2^
Smoking status (n, %)				
*No smoker*	31 (26.3)	11 (30.6)	20 (24.4)	0.744^1^
*Ex smoker*	78 (66.1)	22 (61.1)	56 (68.3)	
*Current smoker*	9 (7.6)	3 (8.3)	6 (7.3)	
FVC (most recent, %; mean, SD)	71.2 (20.2)	79.5 (18.8)	67.3 (19.8)	0.004^2^
DLCO (most recent, %; mean, SD)	41.4 (19.1)	46.0 (17.2)	39.7 (20.1)	0.133^2^
GAP stage (n, %)				
*I*	33 (28.0)	13 (38.2)	20 (26.7)	
*II*	51 (43.2)	15 (44.1)	36 (48.0)	0.425^1^
*III*	25 (21.2)	6 (17.7)	19 (25.3)	
Number of exacerbations (mean, SD)	0.7 (1.0)	0.6 (0.8)	0.8 (1.0)	0.294^2^
Exacerbation (yes; n, %)	55 (46.6)	14 (38.9)	41 (50.0)	0.217^1^
Telomere length ratio (mean, SD)	0.79 (0.35)	0.79 (0.35)	-	-
Antifibrotic therapy (yes; n, %)	44 (37.3)	12 (33.3)	32 (39.0)	0.671^1^

*Abbreviations*: *FVC* Forced Vital Capacity, *DLCO* Diffusing Capacity of the lung for carbon monoxide, *SD* Standard Deviation

*p*-value^a^: assessing differences between patients with/without telomere length ratio measurement

^1^chi-square test

^2^independent samples t-test

In Table 2, personal long-term exposure to outdoor air pollutants, based on geo-coded residential addresses, of all participants as well as of the subgroup are presented. Fifty six percent (56%) of the participants living in the Greater Athens area (a basin surrounding by mountains) were significantly exposed to higher mean NO_2_, PM_2.5_ and PM_10_ concentrations compared to those living outside of it. In contrast the latter were exposed to higher O_3_ concentrations. Exposure to air pollutant concentrations did not differ between patients with or without telomere length ratio measurements. Exposure to PM_10_ and PM_2.5_ did not differ between stable and AE-IPF patients (*p* > 0.05). Stable patients were on average exposed to higher O_3_ concentrations (*p* = 0.026) and lower NO_2_ concentrations (*p* = 0.014) vs AE-IPF patients.

**Table 2:** Distribution of personal long-term exposure to outdoor air pollutants, based on geo-coded residential addresses for all patients and subgroups

**Personal long-term exposure to:**	**Mean value (SD)** ***Min—Max***						***p*-value**
	**All patients** **(*n* = 118)**	**Residing in the Greater Athens Area (*n* = 66)**	**Living outside of the Greater Athens Area (*n* = 52)**	**Patients with telomere length ratio (*n* = 36)**	**AE-IPF** **(*n* = 55)**	**Stable patients** **(*n* = 63)**	
O_3_ (μg/m^3^)	76.8 (14.7) *53.3—105.7*	67.0 (10.4) *53.3 – 93.5*	89.3 (8.4) *67.6 – 105.7*	78.5 (13.8) *53.5 – 102.3*	73.6 (14.4) *53.3 – 100.0*	79.6 (14.5) *55.5 – 105.7*	0.415^1^/ < 0.001^2^
NO_2_ (μg/m^3^)	22.2 (18.6) *0.6—55.8*	36.1 (12.6) *4.7 – 55.8*	4.7 (5.6) *0.6 – 30.2*	20.6 (17.6) *1.1 – 55.8*	26.7 (18.4) *0.9 – 55.8*	18.3 (18.1) *0.6 – 50.8*	0.534 < 0.001^2^
PM_2.5_ (μg/m^3^)	27.2 (7.6) *16.2 – 43.1*	32.2 (6.3) *20.6 – 43.1*	20.7 (2.3) *16.2 – 31.6*	26.5 (7.2) *18.6 – 40.7*	28.5 (7.8) *18.5 – 43.1*	26.0 (7.2) *16.2 – 40.7*	0.519 < 0.001^2^
PM_10_ (μg/m^3^)	30.5 (7.5) *17.9 – 46.6*	35.4 (6.4) *23.3 – 46.6*	24.2 (2.5) *17.9 – 34.6*	29.8 (7.1) *21.8 – 43.8*	31.7 (7.8) *21.0 – 46.6*	29.3 (7.2) *17.9 – 43.9*	0.518 < 0.001^2^

*Abbreviations*: *O*_*3*_ ozone, *PM* particulate matter, *NO*_*2*_ nitrogen dioxide, *SD* Standard Deviation, *AE-IPF* Acute Exacerbation of Idiopathic Pulmonary Fibrosis

*p*-value^1^: independent samples t-test for assessing differences between patients with/without telomere length ratio measurement

*p*-value^2^: independent samples t-test for assessing differences between patients residing in/out of the Greater Athens Area

A positive significant association was detected between AE-IPF and a 10 μg/m^3^ increase in annual mean level of NO_2_ (OR = 1.52, 95%CI:1.15–2.0, *p* = 0.003), PM_2.5_ (OR = 2.21, 95%CI: 1.16–4.20, *p* = 0.016) and PM_10_ (OR = 2.18, 95%CI: 1.15–4.15, *p* = 0.017) independent of age, gender, smoking and lung function impairment and antifibrotic treatment (Table 3). The negative association, we observed, between AE-IPF and long-term average levels of O_3_ was no longer significant after additionally adjusting for long-term exposure to PM_2.5_ concentrations. The results remained practically the same after adjusting for distance to major road (Table S1), mean annual temperature (Table S2) and job (Table S3).

**Table 3:** Long-term personal exposure to concentrations of air pollutants and risk of AE-IPF. Results reported from logistic regression models: Odds Ratio (OR) & 95% C.I., after adjusting for gender, age, smoking habits, recent FVC, recent DLCO and antifibrotic therapy. ^a^ also adjusting for telomere length ratio; ^b^ Ο3: also adjusting for PM_2.5_

**Results reported per (10 μg/m**^**3**^**):**	**All patients**		**Patients residing in the Greater Athens Area (*n* = 66)**		**Patients residing out of the Greater Athens Area (*n* = 52)**	
	**OR** **(95% CI)**	***p*-value**	**OR (95% CI)**	***p*-value**	**OR (95% CI)**	***p*-value**
^1^O_3_	0.60 (0.43 to 0.86)	0.005**	0.57 (0.27 to 1.17)	0.126	0.76 (0.28 to 2.06)	0.590
^**a** 2^O_3_	0.27 (0.07 to 1.00)	0.050				
^**b** 1^O_3_	0.55 (0.27 to 1.13)	0.105	0.42 (0.10 to 1.81)	0.245	0.78 (0.27 to 2.29)	0.654
^**b** 2^O_3_	0.26 (0.01 to 1.05)	0.052				
^1^NO_2_	1.52 (1.15 to 2.00)	0.003**	1.58 (0.88 to 2.87)	0.128	3.89 (0.75 to 20.3)	0.106
^**a** 2^NO_2_	2.64 (0.99 to 7.05)	0.053				
^1^PM_2.5_	2.21 (1.16 to 4.20)	0.016*	2.01 (0.56 to 7.12)	0.282	1.67 (0.08 to 36.5)	0.743
^**a** 2^PM_2.5_	2.78 (0.35 to 22.2)	0.335				
^1^PM_10_	2.18 (1.15 to 4.15)	0.017*	1.96 (0.57 to 6.71)	0.285	1.93 (0.12 to 29.9)	0.637
^**a** 2^PM_10_	2.35 (0.31 to 17.9)	0.410				

*Abbreviations*: *FVC* Forced Vital Capacity, *DLCO* Diffusing Capacity of the lung for carbon monoxide, *O*_*3*_ ozone, *PM* particulate matter, *NO*_*2*_ nitrogen dioxide

^1^All patients (*n* = 118)

^2^All patients with telomere length ratio measurements (*n* = 36)

^*^ statistically significant at a = 5%

^**^ statistically significant at a = 1%

It should be noted that in a sensitivity analysis, after adjustment also for TL in the subgroup of 36 patients, the direction of the associations remained the same as in the whole cohort. However, no statistically significant associations were observed (marginal positive association with NO_2_ and inverse with O_3_) probably due to the small number of patients with measured TL (Table 3).

In Table 4 the distribution of inflammatory mediators between IPF and AE-IPF patients is shown.

**Table 4:** Distribution of inflammatory mediators (pg/mL) in AE-IPF and stable patients

**Inflammatory mediators (pg/mL)**	**Median (IQR)**								
	**Whole cohort (*n* = 93)**			**Patients residing in the Greater Athens Area (*n* = 51)**			**Patients residing out of the Greater Athens Area (*n* = 42)**		
	**All patients** **(*n* = 93)**	**AE-IPF** **(*n* = 49)**	**Stable patients** **(*n* = 44)**	**All patients** **(*n* = 51)**	**AE-IPF** **(*n* = 32)**	**Stable patients** **(*n* = 19)**	**All patients** **(*n* = 42)**	**AE-IPF** **(*n* = 22)**	**Stable patients** **(*n* = 20)**
IL-1a	3.2 (24.3)	3.2 (38.3)	3.2 (0.0)	3.2 (36.6)	3.2 (102.4)	3.2 (0)	3.2 (24.4)	3.2 (17)	3.2 (23.8)
IL-1b	2.8 (2.6)	3.2 (2.6)	1.3 (2.7)	3.2 (2.5)	3.2 (2.3)	1.4 (2.6)	1.1 (2.7)	0.9 (2.8)	1 (2.7)
IL-4	0.5 (1.1)	2 (11.7)	9.8 (25)	2 (13.3)	1.8 (10.8)	9.8 (29)	9.7 (19.3)	10.9 (19.2)	10.1 (23.5)
IL-5	7.8 (15.8)	2 (2.7)	1.6 (1.5)	1.8 (3.2)	2 (3.1)	1.7 (2.2)	1.6 (2)	1.7 (3.6)	1.5 (1.2)
IL-6	1.7 (2.2)	4.3 (9.7)	1.6 (2.4)	3.3 (8.9)	4.3 (13.9)	2.1 (3.4)	2.4 (4.5)	3.9 (6.6)	1.5 (3.4)
IL-8	2.8 (5.2)	10.9 (16.1)	5.1 (5.8)	7.3 (10.5)	10.9 17.3)	5.4 (5.3)	6.5 (8.9)	10.9 (16.5)	4.7 (5.4)
IL-10	7.0 (9.4)	4.7 (8.9)	4.0 (7.3)	4.4 (6.8)	4.5 (8.1)	4 (6.5)	4.5 (10.1)	5.8 (13.8)	3.9 (7.4)
IL-13	4.4 (8.1)	2.1 (5.4)	0.2 (2.0)	1.1 (5.4)	1.7 (5.2)	0.6 (8.8)	0.3 (2.6)	2.7 (11.4)	0.2 (1.3)
MCP-1	338.6 (186.6)	359 (232)	311 (169)	324 (209)	316 (232)	328 (177)	3701 (185)	432 (2601)	288 (185)
MIP-1a	16.0 (23.7)	17.5 (25.7)	10.4 (15.7)	16.9 (26.9)	18.1 (29.2)	11.4 (16.5)	12.2 (20.6)	12.9 (26.2)	9.9 (18.3)
MIP-4	555.0 (268.5)	543 (361)	564 (248)	615 (352)	608 (495)	593 (254)	504 (209)	502 (227)	527 (235)
MMP1	1691 (2189)	1998 (3459)	1185 (1746)	1878 (2711)	1938 (3394)	1690 (2306)	1476 (2053)	2032 (3414)	1067 (1192)
MMP7	2875 (8070)	680 (7644)	8156 (8229)	548 (8035)	548 (6729)	8227 (8612)	7904 (8070)	4755 (7690)	8084 (8097)
MMP9	71,941 (50,498)	74,697 (45,764)	61,817 (44,036)	76,638 (60,233)	74,331 (67,302)	86,489 (60,936)	59,883 (39,402)	75,441 (43,656)	57,949.5 (30,739.2)
TNFa	3.9 (5.1)	3.9 (5.5)	3.6 (5.2)	4 (4.7)	3.9 (4.1)	5.1 (4.7)	3.4 (7.2)	6.1 (7.9)	2.6 (3.9)
TGFb1	6847 (19,363)	7148 (24,161)	6202 (15,300)	7148 (23,122)	7226 (24,052)	5583 (12,605)	5990 (19,013)	6292 (29,717)	6481 (15,844)
OPN	25,581 (46,783)	31,149 (37,819)	23,534 (54,128)	23,539 (50,807)	25,124 (41,382)	15,113 (60,563)	27,263 (40,751)	39,117 (40,684)	25,620 (69,001)

*AE-IPF* Acute Exacerbation of Idiopathic Pulmonary Fibrosis, *IQR* Inter-quartile range

**Fig. 1:**
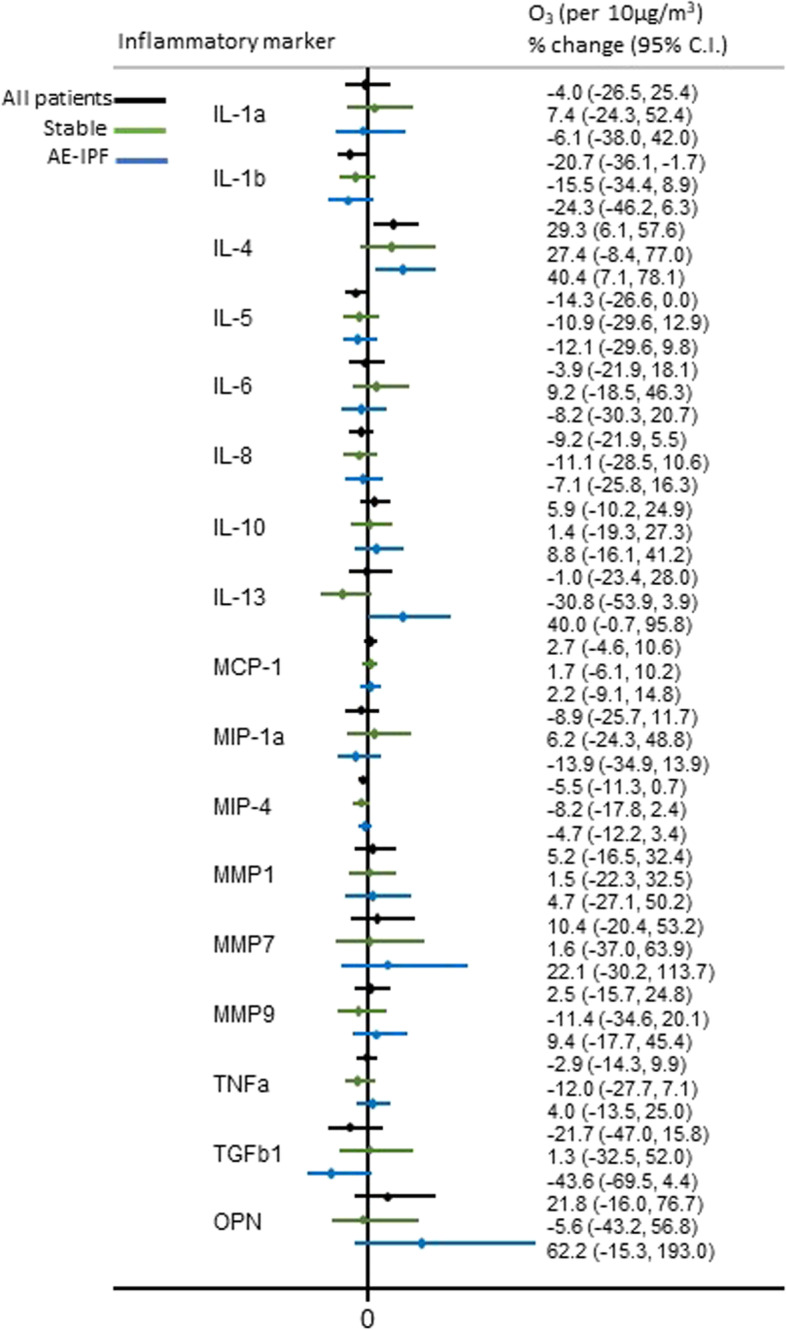
Association of long-term personal exposure to O_3_ and inflammatory mediators (dependent variable is log-transformed), after adjusting for age, sex, smoking status and antifibrotic therapy in all, stable and AE-IPF patients

**Fig. 2:**
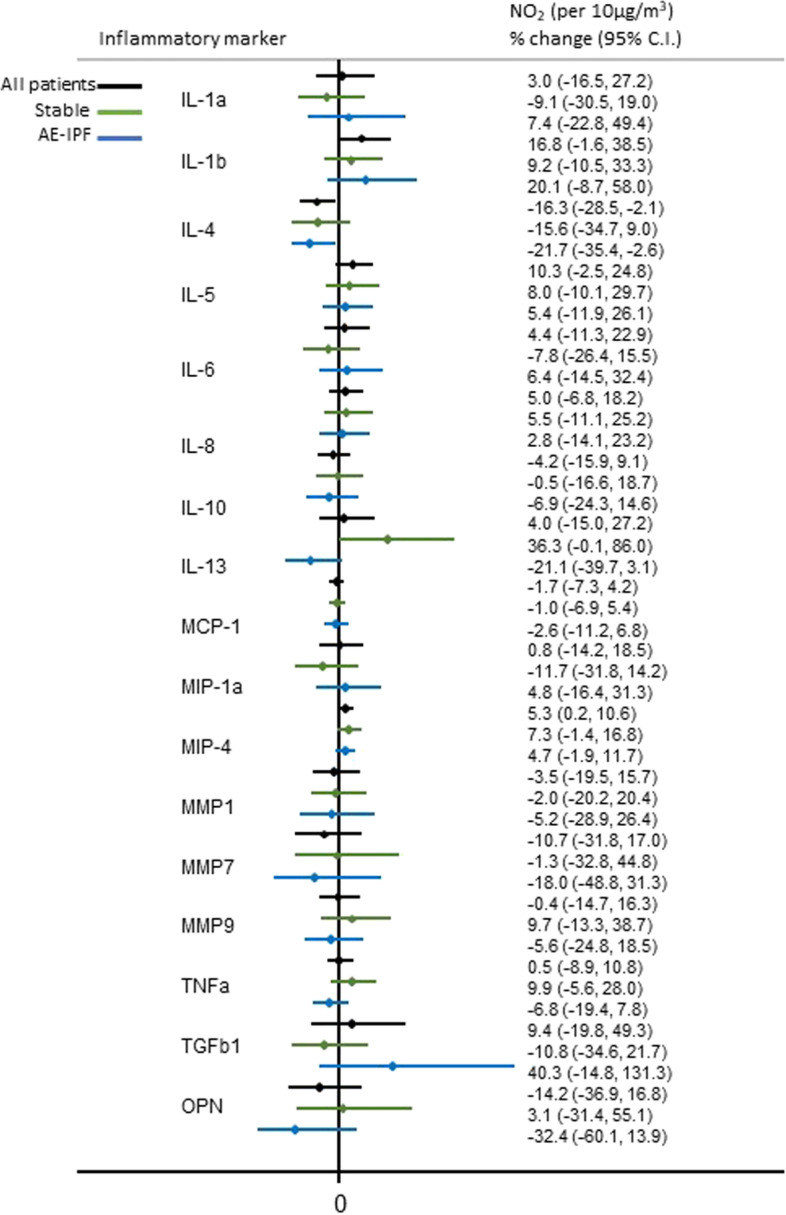
Association of long-term personal exposure to NO_2_ and inflammatory mediators (dependent variable is log-transformed), after adjusting for age, sex, smoking status and antifibrotic therapy in all, stable and AE-IPF patients

**Fig. 3:**
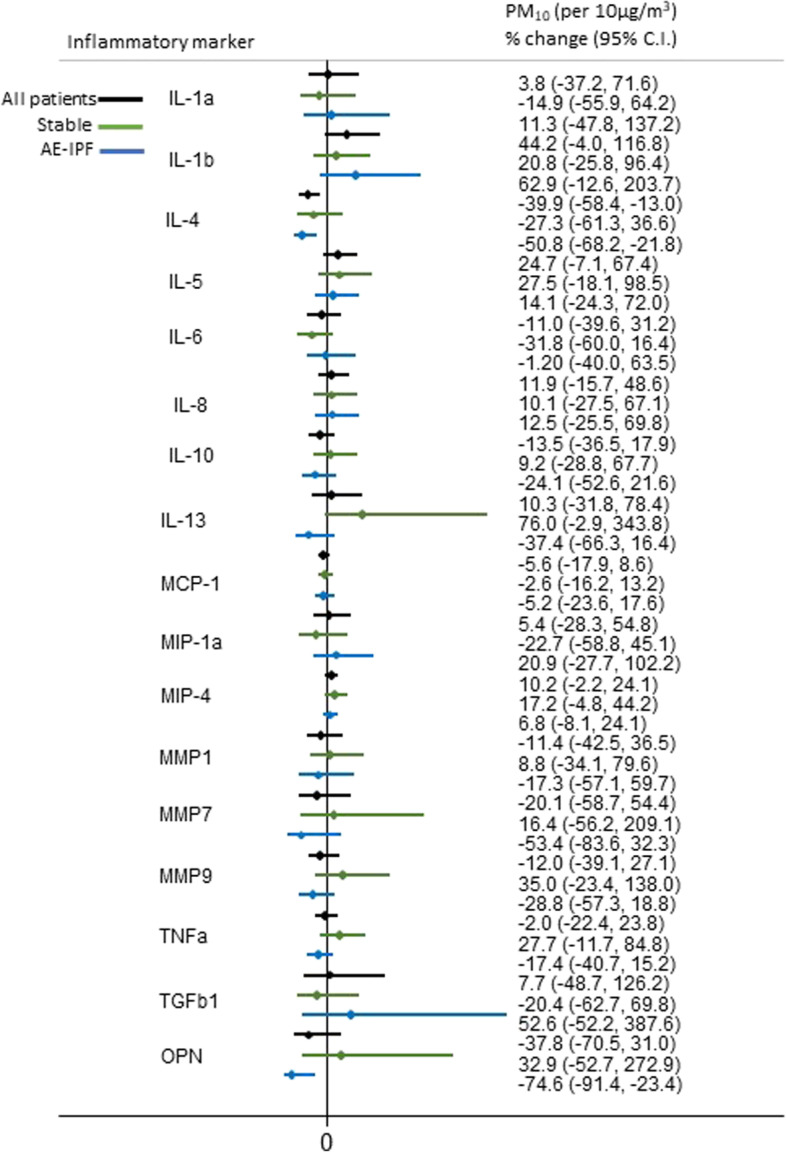
Association of long-term personal exposure to PM_10_ and inflammatory mediators (dependent variable is log-transformed), after adjusting for age, sex, smoking status and antifibrotic therapy in stable and AE-IPF patients

**Fig. 4:**
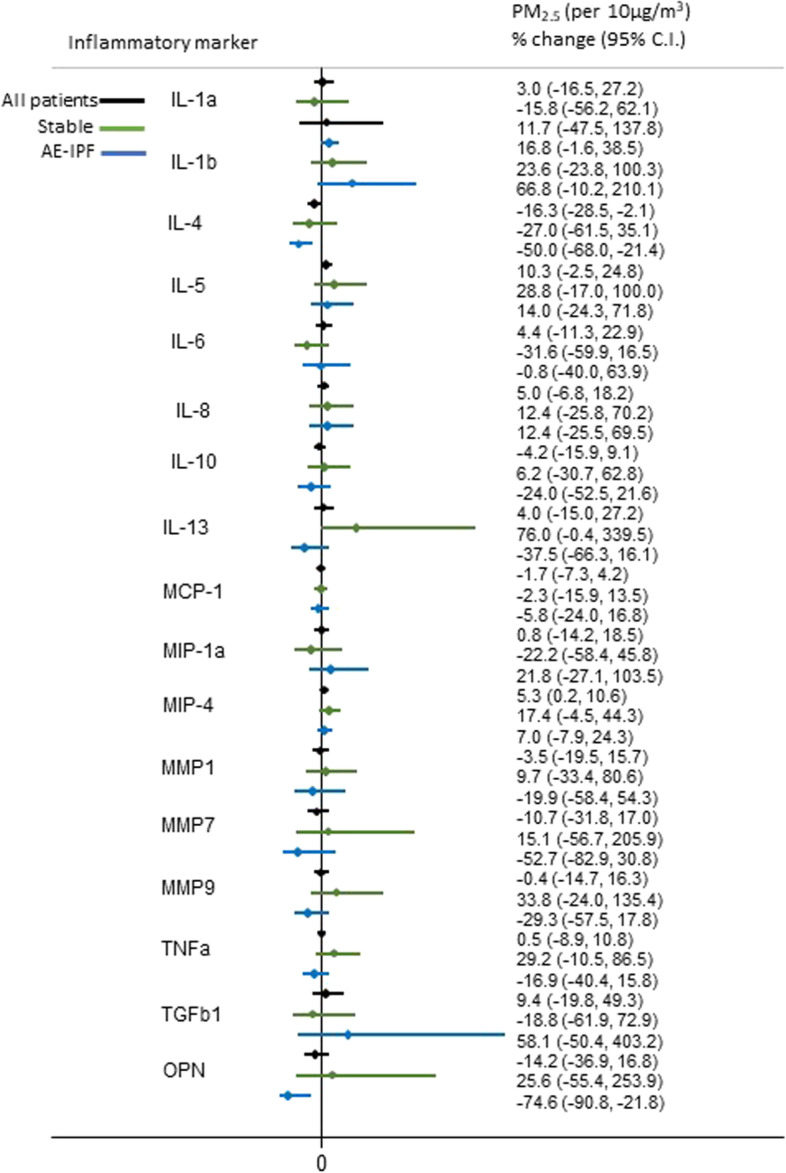
Association of long-term personal exposure to PM_2.5_ and inflammatory mediators (dependent variable is log-transformed), after adjusting for age, sex, smoking status and antifibrotic therapy in all, stable and AE-IPF patients

A 10 μg/m^3^ increase in previous year mean level of O_3_ was significantly positively associated with %change of IL-4 (*p* = 0.014) and marginally with %changes of IL-13 (*p* = 0.059) and osteopontin (*p* = 0.095) in AE-IPF patients. On the contrary, PM_2.5_, PM_10_ and NO_2_ were inversely associated with %changes of IL-4 (*p* = 0.003, *p* = 0.003, *p* = 0.032, respectively) and osteopontin (*p* = 0.013, *p* = 0.013, *p* = 0.085, respectively). In stable IPF patients, only PM_2.5_ and PM_10_ were marginally positively associated with an increase in the %change of IL-13 (*p* = 0.069, *p* = 0.063, respectively) (Figs. 1, 2, 3 and 4). Application of the Bonferroni correction resulted in non-significant effect estimates (Tables S4.1-S4.4).

## Discussion

In this study we observed significant associations between long-term personal exposure to increased levels of NO_2_, PM_2.5_ and PM_10_ and the risk for AE-IPF independent of age, lung function impairment, anti-fibrotic treatment and smoking status. Moreover, out of a number of inflammatory mediators, known to be involved in AE-IPF pathogenesis, that we examined in the peripheral blood, we detected the following in AE-IPF patients: 1) changes in IL-4 positively associated with an increase in long-term personal exposure to O_3_ but inversely with an increase in NO_2_, PM_10_ or PM_2.5_, 2) an indicative positive change in the levels of IL-13 and osteopontin with increased O_3_ and 3) an inverse association between changes in osteopontin and increased exposure in particles (PM_10_ or PM_2.5_)_._ As for stable IPF patients an indicative positive change in IL-13 with increased long-term personal exposure to NO_2_, PM_10_ or PM_2.5_ was also noticed.

According to our results air pollution may represent a potential environmental trigger factor which through repetitive lung injury and induction of lung repair mechanisms and fibrotic process could contribute to the appearance of AE-IPF aggravating thus the outcome in IPF patients.

Ozone at ground level represents a harmful air pollutant, capable to induce oxidative stress and acute airway inflammation [29] while lately long-term ozone exposure has been associated with the appearance of acute respiratory distress syndrome (ARDS), a syndrome that shares many features with AE-IPF [30]. Also, particulate matters (PMs) characterized by the ability to deeply deposit in the respiratory tract induce free radical reactions and inflammation, mechanisms involved in the deterioration and progression of IPF [11, 15]. Over the last years, several studies examined the impact of ambient air pollution exposure in IPF progress [4–13]. In a first study, Johannson et al. reported an increased risk of AE-IPF associated with over the preceding six weeks exposure to increased concentrations of O_3_ and NO_2_ after adjustment for smoking and FVC [4]. Few years later, in another study, they reported associations between lower lung function but not week-to-week changes in lung function with increased exposures to PM_10_, PM_2.5_ and NO_2_ in 25 IPF patients followed-up prospectively for up to 40 weeks [6]. The role of short-term exposure to increased levels of ozone as a risk factor for AE-IPF was also shown in a French IPF cohort (*n* = 152) [9]. Exposure misclassification is an issue in the above-mentioned studies as they did not rely on personal exposure estimates. Recently, in a study with improved exposure assessment, Yoon et al. demonstrated that increased long-term exposure to NO_2_ increases the risk of mortality especially in elderly IPF male patients [13].

So far, the present study, to the best of our knowledge, is the first to investigate the association between long-term personal exposure to air pollutants and AE-IPF as well as with changes in inflammatory mediators known to be involved in its pathogenesis. Our findings show consistent positive associations between the risk of AE-IPF and a 10 μg/m^3^ increase in previous year personal mean level of NO_2_, PM_10_ or PM_2.5_ independent of age, gender, smoking status, lung function impairment and use of antifibrotic treatment, suggesting that ambient air pollution may represent one of a series of factors that could trigger acute exacerbation in IPF. The inverse association we detected between AE-IPF and long-term exposure to O_3_ became non-significant when PM_2.5_ were introduced in the model. The discordance between our results showing no significant inverse associations between long-term personal exposure to increased O_3_ concentrations with AE-IPF and previous studies showing an effect of short-term exposures to increased levels of O_3_ with AE-IPF could be explained by either a spurious event or the fact that O_3_ varies both spatio-temporally. Ozone concentrations tend to be lower in urban compared with suburban areas. The urban decrement is successfully captured by the exposure assessment used in our study. However, O_3_ also has seasonal and daily variation, which depend on meteorological conditions (mainly due to solar radiation but also other meteorological factors, such as ambient temperature, relative humidity and wind velocity). Epidemiological studies of short-term O_3_ exposure are conducted for the “warm period”, since a summer maximum in urban areas is apparent. Moreover, the O_3_ exposure indicator used in short-term studies is the 8-h maximum value in order to capture daily variability. In the present study an all-year 24-h average O_3_ concentration exposure estimate was applicable. This may have led to the cancellation of the seasonal and daily cycle of O_3_, introduced in short-term exposure studies.

Ambient air pollution increases the morbidity in several chronic diseases through diverse mechanisms, such as inflammation, formation of reactive oxygen species and oxidative stress [21, 31–33]. Another potential mechanism by which air pollution could impact the progress of diverse diseases and that has been lately proposed is through oxidative stress-induced telomere erosion [17]. Our findings show that long-term exposure to O_3_ or PMs is associated with changes in the levels of inflammatory mediators in the peripheral blood of IPF patients, such as IL-4, IL-13 and osteopontin, mediators known to be involved in lung repair mechanisms. Both IL-4 and IL-13 are main type 2 cytokines produced by T helper 2 cells that play a critical role in pulmonary fibrosis [34, 35]. Furthermore, osteopontin has been demonstrated to be one of the most up-regulated genes in IPF lungs, with a potential prognostic value in AE-IPF [36, 37].

The associations we revealed between air pollution exposure and the inflammatory mediators, we examined, represent a grain of the presence of lung repair mechanisms and could reflect a prolonged effect of the repetitive chronic lung injury due to air pollution. The more the inflammatory responses to potential noxious stimuli such as air pollution, are up regulated at stable disease and later on exacerbation, the more the lung injury and fibrotic processes are amplified, aggravating thus the outcome of the patients. Finally, the above-mentioned associations, we found, provide evidence for a biological link increasing thus the likelihood of the association between long-term ambient air pollution exposure and AE-IPF to be causal.

Recently, the potential impact of air pollution exposure on TL has emerged [17]. Oxidative stress represents a potential environmental factor that could lead to accelerating telomeric erosion at each replication cycle and thus, to accelerated cellular biological aging [17]. Indeed, short telomeres are known to express the limited tissue renewal capacity in the lung [22, 23, 25, 26]. Thus, these mechanisms could contribute to premature exhaustion and abnormal alveolar re-epithelialisation that have been already linked with IPF development [38]. Short telomeres are recognized as risk factor for idiopathic pulmonary fibrosis (IPF) [22–24]. In this chronic lung disease characterized by irreversible fibrosis, repetitive causative stimuli, such as air pollution exposure could lead the bronchoalveolar epithelium to be constantly replaced [22]. According to the aforementioned data, we hypothesized that patients with shorter telomeres across our IPF cohort could be more susceptible to air pollution exposure having an increased risk for developing AE-IPF compared with individuals with longer ones suggesting that TL could affect the association of air pollution and AE-IPF representing a modifying factor important in the pathogenesis and progress of IPF. Consequently, in the present study a sensitivity analysis performed in a subgroup of our IPF patients did not reveal any confounding effect between long-term ambient air pollution exposure and AE-IPF by TL.

Our study has the following limitations. First, and most important being the relatively small number of patients that could have restrained us from revealing the potential effect modification of genetics. Also, when we applied the Bonferroni correction in an attempt to assess the impact of multiple comparisons in the investigation of the association of long-term personal exposure to predicted air pollutant concentrations and inflammatory mediators in AE-IPF, it resulted, as expected due to the small sample, in non-significant effect estimates. However, it should be noticed that our intention was to delineate underlying mechanisms rather than to compare effects between different markers especially when they are indicators of similar underlying biological effects (e.g., tissue repair). Hence, the emphasis is on the indication of effect rather to which marker is more sensitive. Unfortunately, as mentioned above, limited budget refrained us from measuring TL in the whole cohort. Exposure misclassification is a limitation for all air pollution studies that are based on estimated concentrations. However, the exposure assessment method used in our study is a substantial improvement over using i.e. ecological studies or a nearest air quality monitoring station approach [39], since it better captures within and between area air pollution variability. We estimated an annual average exposure at residence addresses, a proxy considered to be representative for an “individualized” long-term exposure [40]. The limited information on past activity patterns limits the accuracy of our estimates. The resulting misclassification, however, is likely to be non-differential with a tendency to reduce elevated ORs towards the null. Third, TL was measured by using real time quantitative PCR (qPCR), a method that has been lately extensively adapted, as it is more easily performed requiring small amounts of DNA [41]. Finally, it should be noticed that ELISA MMP analyses do not prove matrix metalloproteinase activity, as it merely measures presence. In general, our study represents a prospective, single-academic center study, based on a well-selected group of IPF patients that examined long term personal exposure to air pollution and the risk for AE-IPF identifying modifiable risk factors that could limit disease’s morbidity and mortality.

## Conclusions

Long term personal exposure to increased concentrations of air pollutants is associated with increased risk of AE-IPF in IPF patients. Inflammatory mediators implicated in lung repair mechanisms are involved. Identifying potential modifiable risk factors, such as air pollution exposure could represent a critical preventive tool against IPF progression and morbidity. Large-scale studies are needed to confirm our results and elucidate the involved pathogenetic mechanisms.

## Supplementary Information

**Additional file 1: Table S1.** Long-term personal exposure to concentrations of air pollutants and risk of AE-IPF. Results reported from logistic regression models: Odds Ratio (OR) & 95% C.I., after adjusting for gender, age, smoking habits, recent FVC, recent DLCO, antifibrotic therapy and distance to major road. ^a^ also adjusting for telomere length ratio; ^b^ Ο3: also adjusting for PM_2.5_. **Table S2.** Long-term personal exposure to concentrations of air pollutants and risk of AE-IPF. Results reported from logistic regression models: Odds Ratio (OR) & 95% C.I., after adjusting for gender, age, smoking habits, recent FVC, recent DLCO, antifibrotic therapy and long-term temperature. ^a^ also adjusting for telomere length ratio; ^b^ Ο3: also adjusting for PM_2.5_. **Table S3.** Long-term personal exposure to concentrations of air pollutants and risk of AE-IPF. Results reported from logistic regression models: Odds Ratio (OR) & 95% C.I., after adjusting for gender, age, smoking habits, recent FVC, recent DLCO, antifibrotic therapy and job (blue vs white collar). ^a^ also adjusting for telomere length ratio; ^b^ Ο3: also adjusting for PM_2.5_. **Figure S1.** Map of the participant’s residences and personal exposure to long-term air pollutant concentrations in Greece (air pollution exposure available at http://mapsportal.ypen.gr/maps/?limit=20&offset=0&category__identifier__in=environment%2Fatmosphere). **Table S4.1.** Association* of long-term personal exposure to O_3_ (per 10μg/m^3^) and mediators (dependent variable is log-transformed), after adjusting for age, sex, smoking status and antifibrotics in all patients, stable and AE-IPF patients. **Table S4.2.** Association* of long-term personal exposure to NO_2_ (per 10μg/m^3^) and mediators (dependent variable is log-transformed), after adjusting for age, sex, smoking status and antifibrotics in all patients, stable and AE-IPF patients. **Table S4.3.** Association* of long-term personal exposure to PM_10_ (per 10μg/m^3^) and mediators (dependent variable is log-transformed), after adjusting for age, sex, smoking status and antifibrotics in all patients, stable and AE-IPF patients. **Table S4.4.** Association* of long-term personal exposure to PM_2.5_ (per 10μg/m^3^) and mediators (dependent variable is log-transformed), after adjusting for age, sex, smoking status and antifibrotics in all patients, stable and AE-IPF patients.

## Abbreviations

IPF: Idiopathic pulmonary fibrosis
AE-IPF: Acute Exacerbation of idiopathic pulmonary fibrosis
TL: Telomere length
O_3_: Ozone
NO_2_: Nitrogen dioxide
PM_2.5_: Particles with a 50% cut-off aerodynamic diameter of 2.5 mm
PM_10_: Particles with a 50% cut-off aerodynamic diameter of 10 mm
DAD: Diffuse alveolar damage
UIP: Usual interstitial pneumonia
CT: Computed tomography
CTPA: Computed tomography pulmonary angiogram
BAL: Bronchoalveolar lavage
IL-: Interleukin
CCL-: C–C motif chemokine ligand
MMPs: Matrix metalloproteinases
ELISA: Enzyme-linked immunoabsorbent assay
qPCR: Quantitative polymerase chain reaction
FVC: Forced vital capacity
DLCO: Diffusing capacity for carbon monoxide
ARDS: Acute respiratory distress syndrome
OR: Odds Ratios
C.I.: Confidence Interval
