# Supplementary material for: Long-term personal air pollution exposure and risk for acute exacerbation of idiopathic pulmonary fibrosis
Source: Environ Health. 2021 Aug 30;20:99. doi: 10.1186/s12940-021-00786-z (PMC8406600; doi:10.1186/s12940-021-00786-z)
Supplement: Supplementary file 1 — Additional file 1: Table S1. Long-term personal exposure to concentrations of air pollutants and risk of AE-IPF. Results reported from logistic regression models: Odds Ratio (OR) & 95% C.I., after adjusting for gender, age, smoking habits, recent FVC, recent DLCO, antifibrotic therapy and distance to major road. a also adjusting for telomere length ratio; b Ο3: also adjusting for PM2.5. Table S2. Long-term personal exposure to concentrations of air pollutants and risk of AE-IPF. Results reported from logistic regression models: Odds Ratio (OR) & 95% C.I., after adjusting for gender, age, smoking habits, recent FVC, recent DLCO, antifibrotic therapy and long-term temperature. a also adjusting for telomere length ratio; b Ο3: also adjusting for PM2.5. Table S3. Long-term personal exposure to concentrations of air pollutants and risk of AE-IPF. Results reported from logistic regression models: Odds Ratio (OR) & 95% C.I., after adjusting for gender, age, smoking habits, recent FVC, recent DLCO, antifibrotic therapy and job (blue vs white collar). a also adjusting for telomere length ratio; b Ο3: also adjusting for PM2.5. Figure S1. Map of the participant’s residences and personal exposure to long-term air pollutant concentrations in Greece (air pollution exposure available at http://mapsportal.ypen.gr/maps/?limit=20&offset=0&category__identifier__in=environment%2Fatmosphere). Table S4.1. Association* of long-term personal exposure to O3 (per 10μg/m3) and mediators (dependent variable is log-transformed), after adjusting for age, sex, smoking status and antifibrotics in all patients, stable and AE-IPF patients. Table S4.2. Association* of long-term personal exposure to NO2 (per 10μg/m3) and mediators (dependent variable is log-transformed), after adjusting for age, sex, smoking status and antifibrotics in all patients, stable and AE-IPF patients. Table S4.3. Association* of long-term personal exposure to PM10 (per 10μg/m3) and mediators (dependent variable is log-transfo [file 12940_2021_786_MOESM1_ESM.docx]

**Supplement**

**Long-term personal air pollution exposure and risk for acute exacerbation of idiopathic pulmonary fibrosis**

Ioannis Tomos^1^, Konstantina Dimakopoulou^2^, Effrosyni D. Manali^1^, Spyros A. Papiris^1^, Anna Karakatsani^1^.

^1^2^nd^ Pulmonary Medicine Department, National and Kapodistrian University of Athens, Medical School, “ATTIKON” University Hospital, Haidari, Greece, [etomos@hotmail.com](mailto:etomos@hotmail.com), [fmanali@otenet.gr](mailto:fmanali@otenet.gr), papiris@otenet.gr, akarakats@med.uoa.gr

^2^Department of Hygiene, Epidemiology and Medical Statistics, National and Kapodistrian University of Athens, Medical School, Athens, Greece, kdimakop@med.uoa.gr

**Table S1. Long-term personal exposure to concentrations of air pollutants and risk of AE-IPF. Results reported from logistic regression models: Odds Ratio (OR) & 95% C.I., after adjusting for gender, age, smoking habits, recent FVC, recent DLCO, antifibrotic therapy and distance to major road. ^a^ also adjusting for telomere length ratio; ^b^ Ο3: also adjusting for PM_2.5_.**

| **Results reported per (10μg/m^3^):** | **All patients** | | **Patients residing in the Greater Athens Area (n=66)** | | **Patients residing out of the Greater Athens Area (n=52)** | |
| --- | --- | --- | --- | --- | --- | --- |
|  | **OR**  **(95 % CI)** | **p-value** | **OR**  **(95 % CI)** | **p-value** | **OR**  **(95 % CI)** | **p-value** |
| ^1^O_3_ | 0.60  (0.42 to 0.87) | 0.007** | 0.32  (0.11 to 0.93) | 0.036* | 0.73  (0.27 to 1.99) | 0.539 |
| **^a^** ^2^O_3_ | 0.25  (0.06 to 1.02) | 0.053 |  | | | |
| **^b^** ^1^O_3_ | 0.49  (0.23 to 1.04) | 0.064 | 0.21  (0.03 to 1.45) | 0.113 | 0.74  (0.25 to 2.19) | 0.590 |
| **^b^** ^2^O_3_ | 0.27  (0.02 to 1.04) | 0.052 |  |  |  |  |
| ^1^NO_2_ | 1.49  (1.12 to 1.99) | 0.006** | 2.51  (1.05 to 5.98) | 0.039* | 3.64  (0.70 to 18.9) | 0.124 |
| **^a^** ^2^NO_2_ | 2.66  (0.98 to 7.24) | 0.055 |  | | | |
| ^1^PM_2.5_ | 2.10  (1.06 to 4.15) | 0.033* | 3.90  (0.76 to 20.0) | 0.103 | 1.64  (0.07 to 38.2) | 0.759 |
| **^a^** ^2^PM_2.5_ | 3.01  (0.35 to 25.7) | 0.313 |  | | | |
| ^1^PM_10_ | 2.10  (1.06 to 4.15) | 0.034* | 3.83  (0.77 to 19.1) | 0.101 | 1.93  (0.12 to 32.1) | 0.647 |
| **^a^** ^2^PM_10_ | 2.57  (0.31 to 21.2) | 0.380 |  | | | |

Abbreviations: FVC: Forced Vital Capacity, DLCO: Diffusing Capacity of the lung for carbon monoxide; O_3_: ozone; PM: particulate matter; NO_2_: nitrogen dioxide.

^1^All patients (n= 118)

^2^All patients with telomere length ratio measurements (n=36)

*statistically significant at a=5%

** statistically significant at a=1%

**Table S2. Long-term personal exposure to concentrations of air pollutants and risk of AE-IPF. Results reported from logistic regression models: Odds Ratio (OR) & 95% C.I., after adjusting for gender, age, smoking habits, recent FVC, recent DLCO, antifibrotic therapy and long-term temperature. ^a^ also adjusting for telomere length ratio; ^b^ Ο3: also adjusting for PM_2.5_.**

| **Results reported per (10μg/m^3^):** | **All patients** | | **Patients residing in the Greater Athens Area (n=66)** | | **Patients residing out of the Greater Athens Area (n=52)** | |
| --- | --- | --- | --- | --- | --- | --- |
|  | **OR**  **(95 % CI)** | **p-value** | **OR**  **(95 % CI)** | **p-value** | **OR**  **(95 % CI)** | **p-value** |
| ^1^O_3_ | 0.66  (0.46 to 0.94) | 0.022* | 0.61  (0.30 to 1.27) | 0.189 | 0.49  (0.15 to 1.56) | 0.229 |
| **^a^** ^2^O_3_ | 0.28  (0.07 to 1.16) | 0.079 |  | | | |
| **^b^** ^1^O_3_ | 0.54  (0.26 to 1.11) | 0.094 | 0.83  (0.16 to 4.31) | 0.829 | 0.41  (0.11 to 1.56) | 0.194 |
| **^b^** ^2^O_3_ | 0.21  (0.02 to 1.49) | 0.069 |  |  |  |  |
| ^1^NO_2_ | 1.39  (1.04 to 1.86) | 0.028* | 1.46  (0.81 to 2.64) | 0.205 | 3.59  (0.60 to 21.4) | 0.162 |
| **^a^** ^2^NO_2_ | 2.36  (0.83 to 6.70) | 0.106 |  | | | |
| ^1^PM_2.5_ | 1.81  (0.93 to 3.52) | 0.081 | 2.50  (0.66 to 9.51) | 0.179 | 1.24  (0.03 to 54.8) | 0.911 |
| **^a^** ^2^PM_2.5_ | 2.19  (0.19 to 25.2) | 0.528 |  | | | |
| ^1^PM_10_ | 1.77  (0.91 to 3.45) | 0.093 | 2.37  (0.65 to 8.61) | 0.190 | 1.18  (0.04 to 31.3) | 0.920 |
| **^a^** ^2^PM_10_ | 1.81  (0.15 to 21.4) | 0.638 |  | | | |

Abbreviations: FVC: Forced Vital Capacity, DLCO: Diffusing Capacity of the lung for carbon monoxide; O_3_: ozone; PM: particulate matter; NO_2_: nitrogen dioxide.

^1^All patients (n= 118)

^2^All patients with telomere length ratio measurements (n=36)

*statistically significant at a=5%

** statistically significant at a=1%

**Table S3. Long-term personal exposure to concentrations of air pollutants and risk of AE-IPF. Results reported from logistic regression models: Odds Ratio (OR) & 95% C.I., after adjusting for gender, age, smoking habits, recent FVC, recent DLCO, antifibrotic therapy and job (blue vs white collar). ^a^ also adjusting for telomere length ratio; ^b^ Ο3: also adjusting for PM_2.5_.**

| **Results reported per (10μg/m^3^):** | **All patients** | | **Patients residing in the Greater Athens Area (n=59)** | | **Patients residing out of the Greater Athens Area (n=45)** | |
| --- | --- | --- | --- | --- | --- | --- |
|  | **OR**  **(95 % CI)** | **p-value** | **OR**  **(95 % CI)** | **p-value** | **OR**  **(95 % CI)** | **p-value** |
| ^1^O_3_ | 0.67  (0.46 to 0.97) | 0.036* | 0.46  (0.20 to 1.09) | 0.078 | 1.17  (0.39 to 3.52) | 0.780 |
| **^a^** ^2^O_3_ | 0.42  (0.09 to 1.88) | 0.257 |  | | | |
| **^b^** ^1^O_3_ | 0.66  (0.32 to 1.36) | 0.262 | 0.35  (0.07 to 1.68) | 0.191 | 0.96  (0.30 to 3.00) | 0.939 |
| **^b^** ^2^O_3_ | 0.81  (0.52 to 1.28) | 0.375 |  |  |  |  |
| ^1^NO_2_ | 1.43  (1.06 to 1.93) | 0.020* | 1.88  (0.93 to 3.78) | 0.079 | 2.62  (0.39 to 17.6) | 0.321 |
| **^a^** ^2^NO_2_ | 2.10  (0.63 to 6.97) | 0.228 |  | | | |
| ^1^PM_2.5_ | 1.93  (0.94 to 3.95) | 0.073 | 2.53  (0.59 to 10.9) | 0.211 | 1.01  (0.07 to 13.8) | 0.201 |
| **^a^** ^2^PM_2.5_ | 1.67  (0.14 to 20.23) | 0.686 |  | | | |
| ^1^PM_10_ | 1.90  (0.92 to 3.90) | 0.081 | 2.46  (0.60 to 10.1) | 0.213 | 1.60  (0.02 to 11.3) | 0.288 |
| **^a^** ^2^PM_10_ | 1.45  (0.11 to 18.6) | 0.777 |  | | | |

Abbreviations: FVC: Forced Vital Capacity, DLCO: Diffusing Capacity of the lung for carbon monoxide; O_3_: ozone; PM: particulate matter; NO_2_: nitrogen dioxide.

^1^All patients (n= 104)

^2^All patients with telomere length ratio measurements (n=28)

*statistically significant at a=5%

** statistically significant at a=1%

**Table S3. Long-term personal exposure to concentrations of air pollutants and risk of AE-IPF. Results reported from logistic regression models: Odds Ratio (OR) & 95% C.I., after adjusting for gender, age, smoking habits, recent FVC, recent DLCO, antifibrotic therapy and job (blue vs white collar). ^a^ also adjusting for telomere length ratio; ^b^ Ο3: also adjusting for PM_2.5_.**

| **Results reported per (10μg/m^3^):** | **All patients** | | **Patients residing in the Greater Athens Area (n=59)** | | **Patients residing out of the Greater Athens Area (n=45)** | |
| --- | --- | --- | --- | --- | --- | --- |
|  | **OR**  **(95 % CI)** | **p-value** | **OR**  **(95 % CI)** | **p-value** | **OR**  **(95 % CI)** | **p-value** |
| ^1^O_3_ | 0.67  (0.46 to 0.97) | 0.036* | 0.46  (0.20 to 1.09) | 0.078 | 1.17  (0.39 to 3.52) | 0.780 |
| **^a^** ^2^O_3_ | 0.42  (0.09 to 1.88) | 0.257 |  | | | |
| **^b^** ^1^O_3_ | 0.66  (0.32 to 1.36) | 0.262 | 0.35  (0.07 to 1.68) | 0.191 | 0.96  (0.30 to 3.00) | 0.939 |
| **^b^** ^2^O_3_ | 0.81  (0.52 to 1.28) | 0.375 |  |  |  |  |
| ^1^NO_2_ | 1.43  (1.06 to 1.93) | 0.020* | 1.88  (0.93 to 3.78) | 0.079 | 2.62  (0.39 to 17.6) | 0.321 |
| **^a^** ^2^NO_2_ | 2.10  (0.63 to 6.97) | 0.228 |  | | | |
| ^1^PM_2.5_ | 1.93  (0.94 to 3.95) | 0.073 | 2.53  (0.59 to 10.9) | 0.211 | 1.01  (0.07 to 13.8) | 0.201 |
| **^a^** ^2^PM_2.5_ | 1.67  (0.14 to 20.23) | 0.686 |  | | | |
| ^1^PM_10_ | 1.90  (0.92 to 3.90) | 0.081 | 2.46  (0.60 to 10.1) | 0.213 | 1.60  (0.02 to 11.3) | 0.288 |
| **^a^** ^2^PM_10_ | 1.45  (0.11 to 18.6) | 0.777 |  | | | |

Abbreviations: FVC: Forced Vital Capacity, DLCO: Diffusing Capacity of the lung for carbon monoxide; O_3_: ozone; PM: particulate matter; NO_2_: nitrogen dioxide.

^1^All patients (n= 104)

^2^All patients with telomere length ratio measurements (n=28)

*statistically significant at a=5%

** statistically significant at a=1%

Figure S1. Map of the participant’s residences and personal exposure to long-term air pollutant concentrations in Greece (air pollution exposure available at <http://mapsportal.ypen.gr/maps/?limit=20&offset=0&category__identifier__in=environment%2Fatmosphere>).


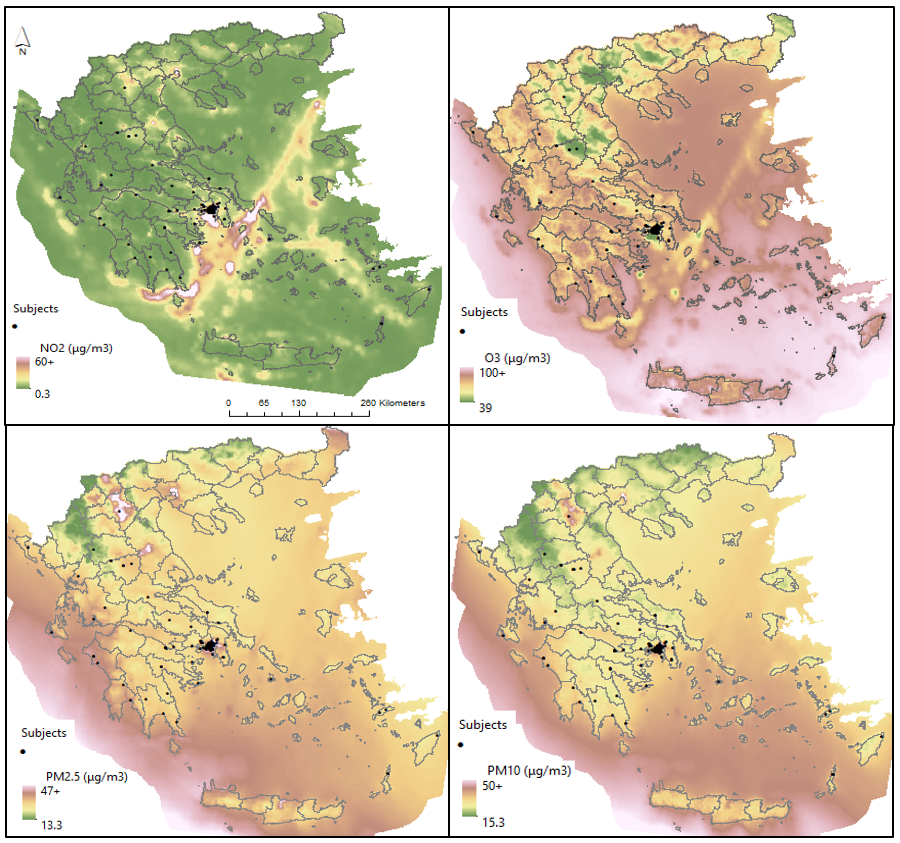


**Table S4.1. Association* of long-term personal exposure to O_3_ (per 10μg/m^3^) and mediators (dependent variable is log-transformed), after adjusting for age, sex, smoking status and antifibrotics in all patients, stable and AE-IPF patients.**

| **Inflammatory Marker** | **All patients** | | | **Stable patients** | | | **AE-IPF patients** | | |
| --- | --- | --- | --- | --- | --- | --- | --- | --- | --- |
|  | **% change** | **95% C.I.** | **p** | **% change** | **95% C.I.** | **p** | **% change** | **95% C.I.** | **p** |
| IL-1a | -4.0 | (-26.5,25.4) | 0.762 | 7.4 | (-24.3,52.4) | 0.808 | -6.1 | (-38,42) | 0.760 |
| IL-1b | -20.7 | (-36.1,-1.7) | 0.035 | -15.5 | (-34.4,8.9) | 0.175 | -24.3 | (-46.2,6.3) | 0.105 |
| IL-4 | 29.3 | (6.1,57.6) | 0.014 | 27.4 | (-8.4,77) | 0.816 | 40.4 | (7.1,78.1) | 0.014 |
| IL-5 | -14.3 | (-26.6,0) | 0.059 | -10.9 | (-29.6,12.9) | 0.551 | -12.1 | (-29.6,9.8) | 0.247 |
| IL-6 | -3.9 | (-21.9,18.1) | 0.700 | 9.2 | (-18.5,46.3) | 0.281 | -8.2 | (-30.3,20.7) | 0.530 |
| IL-8 | -9.2 | (-21.9,5.5) | 0.203 | -11.1 | (-28.5,10.6) | 0.134 | -7.1 | (-25.8,16.3) | 0.512 |
| IL-10 | 5.9 | (-10.2,24.9) | 0.493 | 1.4 | (-19.3,27.3) | 0.554 | 8.8 | (-16.1,41.2) | 0.516 |
| IL-13 | -1.0 | (-23.4,28) | 0.940 | -30.8 | (-53.9,3.9) | 0.821 | 40.0 | (-0.7,95.8) | 0.059 |
| MCP-1 | 2.7 | (-4.6,10.6) | 0.473 | 1.7 | (-6.1,10.2) | 0.697 | 2.2 | (-9.1,14.8) | 0.711 |
| MIP-1a | -8.9 | (-25.7,11.7) | 0.368 | 6.2 | (-24.3,48.8) | 0.524 | -13.9 | (-34.9,13.9) | 0.286 |
| MIP-4 | -5.5 | (-11.3,0.7) | 0.079 | -8.2 | (-17.8,2.4) | 0.988 | -4.7 | (-12.2,3.4) | 0.241 |
| MMP1 | 5.2 | (-16.5,32.4) | 0.663 | 1.5 | (-22.3,32.5) | 0.97 | 4.7 | (-27.1,50.2) | 0.801 |
| MMP7 | 10.4 | (-20.4,53.2) | 0.542 | 1.6 | (-37,63.9) | 0.91 | 22.1 | (-30.2,113.7) | 0.460 |
| MMP9 | 2.5 | (-15.7,24.8) | 0.8 | -11.4 | (-34.6,20.1) | 0.457 | 9.4 | (-17.7,45.4) | 0.528 |
| TNFa | -2.9 | (-14.3,9.9) | 0.635 | -12.0 | (-27.7,7.1) | 0.715 | 4.0 | (-13.5,25) | 0.672 |
| TGFb1 | -21.7 | (-47,15.8) | 0.217 | 1.3 | (-32.5,52) | 0.857 | -43.6 | (-69.5,4.4) | 0.067 |
| OPN | 21.8 | (-16,76.7) | 0.095 | -5.6 | (-43.2,56.8) | 0.981 | 62.2 | (-15.3,193) | 0.095 |

***After applying Bonferroni correction for multiple comparisons, a result was considered as statistically significant if p-value< 0.003.**

**Table S4.2. Association* of long-term personal exposure to NO_2_ (per 10μg/m^3^) and mediators (dependent variable is log-transformed), after adjusting for age, sex, smoking status and antifibrotics in all patients, stable and AE-IPF patients.**

| **Inflammatory Marker** | **All patients** | | | **Stable patients** | | | **AE-IPF patients** | | |
| --- | --- | --- | --- | --- | --- | --- | --- | --- | --- |
|  | **% change** | **95% C.I.** | **p** | **% change** | **95% C.I.** | **p** | **% change** | **95% C.I.** | **p** |
| IL-1a | 21.3 | (-16.5,27.2) | 0.779 | -9.1 | (-30.5,19) | 0.477 | 7.4 | (-22.8,49.4) | 0.414 |
| IL-1b | 28.9 | (-1.6,38.5) | 0.075 | 9.2 | (-10.5,33.3) | 0.375 | 20.1 | (-8.7,58) | 0.105 |
| IL-4 | -30.0 | (-28.5,-2.1) | 0.027 | -15.6 | (-34.7,9) | 0.185 | -21.7 | (-35.4,-2.6) | 0.032 |
| IL-5 | 34.3 | (-2.5,24.8) | 0.116 | 8.0 | (-10.1,29.7) | 0.398 | 5.4 | (-11.9,26.1) | 0.555 |
| IL-6 | 9.1 | (-11.3,22.9) | 0.599 | -7.8 | (-26.4,15.5) | 0.469 | 6.4 | (-14.5,32.4) | 0.655 |
| IL-8 | 25.8 | (-6.8,18.2) | 0.418 | 5.5 | (-11.1,25.2) | 0.526 | 2.8 | (-14.1,23.2) | 0.158 |
| IL-10 | 13.8 | (-15.9,9.1) | 0.516 | -0.5 | (-16.6,18.7) | 0.952 | -6.9 | (-24.3,14.6) | 0.287 |
| IL-13 | -11.6 | (-15,27.2) | 0.703 | 36.3 | (-0.1,86) | 0.051 | -21.1 | (-39.7,3.1) | 0.111 |
| MCP-1 | -0.7 | (-7.3,4.2) | 0.554 | -1.0 | (-6.9,5.4) | 0.751 | -2.6 | (-11.2,6.8) | 0.911 |
| MIP-1a | 1.5 | (-14.2,18.5) | 0.921 | -11.7 | (-31.8,14.2) | 0.330 | 4.8 | (-16.4,31.3) | 0.931 |
| MIP-4 | 4.3 | (0.2,10.6) | 0.041 | 7.3 | (-1.4,16.8) | 0.099 | 4.7 | (-1.9,11.7) | 0.102 |
| MMP1 | 18.9 | (-19.5,15.7) | 0.699 | -2.0 | (-20.2,20.4) | 0.844 | -5.2 | (-28.9,26.4) | 0.454 |
| MMP7 | 6.8 | (-31.8,17) | 0.402 | -1.3 | (-32.8,44.8) | 0.941 | -18.0 | (-48.8,31.3) | 0.816 |
| MMP9 | -4.0 | (-14.7,16.3) | 0.961 | 9.7 | (-13.3,38.7) | 0.427 | -5.6 | (-24.8,18.5) | 0.829 |
| TNFa | -2.1 | (-8.9,10.8) | 0.919 | 9.9 | (-5.6,28) | 0.216 | -6.8 | (-19.4,7.8) | 0.235 |
| TGFb1 | 28.3 | (-19.8,49.3) | 0.565 | -10.8 | (-34.6,21.7) | 0.458 | 40.3 | (-14.8,131.3) | 0.502 |
| OPN | 22.6 | (-36.9,16.8) | 0.322 | 3.1 | (-31.4,55.1) | 0.875 | -32.4 | (-60.1,13.9) | 0.085 |

***After applying Bonferroni correction for multiple comparisons, a result was considered as statistically significant if p-value< 0.003.**

**Table S4.3. Association* of long-term personal exposure to PM_10_ (per 10μg/m^3^) and mediators (dependent variable is log-transformed), after adjusting for age, sex, smoking status and antifibrotics in all patients, stable and AE-IPF patients.**

| **Inflammatory Marker** | **All patients** | | | **Stable patients** | | | **AE-IPF patients** | | |
| --- | --- | --- | --- | --- | --- | --- | --- | --- | --- |
|  | **% change** | **95% C.I.** | **p** | **% change** | **95% C.I.** | **p** | **% change** | **95% C.I.** | **p** |
| IL-1a | 3.8 | (-37.2,71.6) | 0.883 | -14.9 | (-55.9,64.2) | 0.62 | 11.3 | (-47.8,137.2) | 0.777 |
| IL-1b | 44.2 | (-4,116.8) | 0.077 | 20.8 | (-25.8,96.4) | 0.435 | 62.9 | (-12.6,203.7) | 0.121 |
| IL-4 | -39.9 | (-58.4,-13) | 0.008 | -27.3 | (-61.3,36.6) | 0.31 | -50.8 | (-68.2,-21.8) | 0.003 |
| IL-5 | 24.7 | (-7.1,67.4) | 0.139 | 27.5 | (-18.1,98.5) | 0.271 | 14.1 | (-24.3,72) | 0.521 |
| IL-6 | -11.0 | (-39.6,31.2) | 0.553 | -31.8 | (-60,16.4) | 0.154 | -1.2 | (-40.3,63.5) | 0.963 |
| IL-8 | 11.9 | (-15.7,48.6) | 0.433 | 10.1 | (-27.5,67.1) | 0.643 | 12.5 | (-25.5,69.8) | 0.568 |
| IL-10 | -13.5 | (-36.5,17.9) | 0.335 | 9.2 | (-28.8,67.7) | 0.676 | -24.1 | (-52.6,21.6) | 0.245 |
| IL-13 | 10.3 | (-31.8,78.4) | 0.686 | 76.0 | (-2.9,343.8) | 0.063 | -37.4 | (-66.3,16.4) | 0.135 |
| MCP-1 | -5.6 | (-17.9,8.6) | 0.415 | -2.6 | (-16.2,13.2) | 0.723 | -5.2 | (-23.6,17.6) | 0.62 |
| MIP-1a | 5.4 | (-28.3,54.8) | 0.786 | -22.7 | (-58.8,45.1) | 0.41 | 20.9 | (-27.7,102.2) | 0.461 |
| MIP-4 | 10.2 | (-2.2,24.1) | 0.109 | 17.2 | (-4.8,44.2) | 0.129 | 6.8 | (-8.1,24.1) | 0.381 |
| MMP1 | -11.4 | (-42.5,36.5) | 0.579 | 8.8 | (-34.1,79.6) | 0.733 | -17.3 | (-57.1,59.7) | 0.564 |
| MMP7 | -20.1 | (-58.7,54.4) | 0.494 | 16.4 | (-56.2,209.1) | 0.747 | -53.4 | (-83.6,32.3) | 0.141 |
| MMP9 | -12.0 | (-39.1,27.1) | 0.49 | 35.0 | (-23.4,138) | 0.288 | -28.8 | (-57.3,18.8) | 0.188 |
| TNFa | -2.0 | (-22.4,23.8) | 0.864 | 27.7 | (-11.7,84.8) | 0.186 | -17.4 | (-40.7,15.2) | 0.253 |
| TGFb1 | 7.7 | (-48.7,126.2) | 0.842 | -20.4 | (-62.7,69.8) | 0.543 | 52.6 | (-52.2,387.6) | 0.467 |
| OPN | -37.8 | (-70.5,31) | 0.204 | 32.9 | (-52.7,272.9) | 0.567 | -74.6 | (-91.4,-23.4) | 0.013 |

***After applying Bonferroni correction for multiple comparisons, a result was considered as statistically significant if p-value< 0.003.**

**Table S4.4. Association* of long-term personal exposure to PM_2.5_ (per 10μg/m^3^) and mediators (dependent variable is log-transformed), after adjusting for age, sex, smoking status and antifibrotics in all patients, stable and AE-IPF patients.**

| **Inflammatory Marker** | **All patients** | | | **Stable patients** | | | **AE-IPF patients** | | |
| --- | --- | --- | --- | --- | --- | --- | --- | --- | --- |
|  | **% change** | **95% C.I.** | **p** | **% change** | **95% C.I.** | **p** | **% change** | **95% C.I.** | **p** |
| IL-1a | 3.0 | (-16.5,27.2) | 0.883 | -15.8 | (-56.2,62.1) | 0.777 | 11.7 | (-47.5,137.8) | 0.768 |
| IL-1b | 16.8 | (-1.6,38.5) | 0.077 | 23.6 | (-23.8,100.3) | 0.121 | 66.8 | (-10.2,210.1) | 0.103 |
| IL-4 | -16.3 | (-28.5,-2.1) | 0.008 | -27.0 | (-61.5,35.1) | 0.003 | -50.0 | (-68,-21.4) | 0.003 |
| IL-5 | 10.3 | (-2.5,24.8) | 0.139 | 28.8 | (-17,100) | 0.521 | 14.0 | (-24.3,71.8) | 0.521 |
| IL-6 | 4.4 | (-11.3,22.9) | 0.553 | -31.6 | (-59.9,16.5) | 0.963 | -0.8 | (-40,63.9) | 0.973 |
| IL-8 | 5.0 | (-6.8,18.2) | 0.433 | 12.4 | (-25.8,70.2) | 0.568 | 12.4 | (-25.5,69.5) | 0.57 |
| IL-10 | -4.2 | (-15.9,9.1) | 0.335 | 6.2 | (-30.7,62.8) | 0.245 | -24.0 | (-52.5,21.6) | 0.246 |
| IL-13 | 4.0 | (-15,27.2) | 0.686 | 76.0 | (-0.4,339.5) | 0.069 | -37.5 | (-66.3,16.1) | 0.133 |
| MCP-1 | -1.7 | (-7.3,4.2) | 0.415 | -2.3 | (-15.9,13.5) | 0.62 | -5.8 | (-24,16.8) | 0.578 |
| MIP-1a | 0.8 | (-14.2,18.5) | 0.786 | -22.2 | (-58.4,45.8) | 0.461 | 21.8 | (-27.1,103.5) | 0.443 |
| MIP-4 | 5.3 | (0.2,10.6) | 0.109 | 17.4 | (-4.5,44.3) | 0.381 | 7.0 | (-7.9,24.3) | 0.369 |
| MMP1 | -3.5 | (-19.5,15.7) | 0.579 | 9.7 | (-33.4,80.6) | 0.564 | -19.9 | (-58.4,54.3) | 0.499 |
| MMP7 | -10.7 | (-31.8,17) | 0.494 | 15.1 | (-56.7,205.9) | 0.141 | -52.7 | (-82.9,30.8) | 0.138 |
| MMP9 | -0.4 | (-14.7,16.3) | 0.490 | 33.8 | (-24,135.4) | 0.188 | -29.3 | (-57.5,17.8) | 0.178 |
| TNFa | 0.5 | (-8.9,10.8) | 0.864 | 29.2 | (-10.5,86.5) | 0.253 | -16.9 | (-40.4,15.8) | 0.267 |
| TGFb1 | 9.4 | (-19.8,49.3) | 0.842 | -18.8 | (-61.9,72.9) | 0.467 | 58.1 | (-50.4,403.2) | 0.43 |
| OPN | -14.2 | (-36.9,16.8) | 0.204 | 25.6 | (-55.4,253.9) | 0.418 | -74.6 | (-90.8,-21.8) | 0.013 |

***After applying Bonferroni correction for multiple comparisons, a result was considered as statistically significant if p-value< 0.003.**
